# The Th1/Tfh-like biased responses elicited by the rASP-1 innate adjuvant are dependent on TRIF and Type I IFN receptor pathways

**DOI:** 10.3389/fimmu.2022.961094

**Published:** 2022-09-02

**Authors:** Parakkal Jovvian George, Radu Marches, Djamel Nehar-Belaid, Jacques Banchereau, Sara Lustigman

**Affiliations:** ^1^ Laboratory Molecular Parasitology, Lindsley F. Kimball Research Institute, New York Blood Center, New York, NY, United States; ^2^ The Jackson Laboratory for Genomic Medicine, Farmington, CT, United States

**Keywords:** rASP-1, innate adjuvant, TLR4, TRIF, Type I interferon receptor, conventional DCs, RNA-Seq, influenza vaccine

## Abstract

*Ov*-*ASP-1* (rASP-1), a parasite-derived protein secreted by the helminth *Onchocerca volvulus*, is an adjuvant which enhances the potency of the influenza trivalent vaccine (IIV3), even when used with 40-fold less IIV3. This study is aimed to provide a deeper insight into the molecular networks that underline the adjuvanticity of rASP-1. Here we show that rASP-1 stimulates mouse CD11c^+^ bone marrow-derived dendritic (BMDCs) to secrete elevated levels of IL-12p40, TNF-α, IP-10 and IFN-β in a TRIF-dependent but MyD88-independent manner. rASP-1-activated BMDCs promoted the differentiation of naïve CD4^+^ T cells into Th1 cells (IFN-γ^+^) that was TRIF- and type I interferon receptor (IFNAR)-dependent, and into Tfh-like cells (IL21^+^) and Tfh1 (IFN-γ^+^ IL21^+)^ that were TRIF-, MyD88- and IFNAR-dependent. rASP-1-activated BMDCs promoted the differentiation of naïve CD4^+^ T cells into Th17 (IL-17^+^) cells only when the MyD88 pathway was inhibited. Importantly, rASP-1-activated human blood cDCs expressed upregulated genes that are associated with DC maturation, type I IFN and type II IFN signaling, as well as TLR4-TRIF dependent signaling. These activated cDCs promoted the differentiation of naïve human CD4^+^ T cells into Th1, Tfh-like and Th17 cells. Our data thus confirms that the rASP-1 is a potent innate adjuvant that polarizes the adaptive T cell responses to Th1/Tfh1 in both mouse and human DCs. Notably, the rASP-1-adjuvanted IIV3 vaccine elicited protection of mice from a lethal H1N1 infection that is also dependent on the TLR4-TRIF axis and IFNAR signaling pathway, as well as on its ability to induce anti-IIV3 antibody production.

## Introduction

Vaccines have been traditionally developed to protect the host against infections and/or pathogen-induced diseases (1). However, vaccines are now being developed to also protect against cancer, allergic diseases, and symptomatic autoimmune diseases (2–5). Vaccines against infectious agents typically contain dead, inactivated organisms or purified products derived from them. However, weak vaccines or subunit vaccines typically must contain one or more adjuvants to enhance safely their immunogenicity and/or efficacy in all ages. The ability of adjuvanted vaccine antigens to elicit improved protective immune response(s) was demonstrated a century ago when aluminum salts (alum) were introduced as adjuvants (6). Although alum is widely used in many licensed human vaccines (7), it is not always an optimal adjuvant as some of these adjuvanted vaccines require multiple doses to induce protection, and often they drive Th2- over Th1-polarized immunity (8). Adjuvants mostly function by interacting with innate immune cells, such as dendritic cells (DCs), that in turn activate multiple immune networks thereby regulating the nature, duration, and intensity of protective immune responses (9–14)

The recombinant ASP-1 adjuvant (rASP-1) is derived from the human parasite *Onchocerca volvulus* protein named *Ov*-ASP-1. In mice, it induced a balanced Th1 (IgG2)- and Th2 (IgG1)-associated antibody response, a Th1-biased cytokine response, and protective immune responses when combined with commercial vaccines or with various experimental vaccine antigens (15–17). In mice, the rASP-1-adjuvanted seasonal trivalent influenza vaccine (IIV3) induced protection from a lethal H1N1 infection that was dependent on CD4^+^ T cells, but independent of the production of neutralizing antibodies and MyD88 signaling (17). Initial *in vitro* studies have shown that rASP-1 activates and matures human monocyte-derived DCs (MoDC) (18, 19) as well as upregulates the expression of genes associated with MyD88-independent TLR signaling (17). Our present study is focused on comparing the ability of rASP-1 to activate both mouse and human cDCs and their consequent crosstalk with CD4^+^ T cells. We also show that the rASP-1 adjuvant exerts its activities *in vitro* and *in vivo* through the TLR4-TRIF axis and type I interferon receptor (IFNAR) signaling.

## Materials and methods

### Ethics statement

We used leukopaks collected by the New York Blood Center, Component Laboratory. Gender and ethnic minority distribution of the units of Leukocytes from whom the blood have been collected reflect that of the New York metropolitan area from which NYBC staff are drawn. No special distinction was made of gender or ethnicity. No results or data obtained using these samples has entered any blood donor data bank at the NYBC and donors will not be identified in any research publications arising from the study.

Mice were maintained in AAALAC approved barrier facilities at New York Blood Center (NYBC), and allowed to acclimatize for at least one week in the animal facility prior to use. All protocols involving mice were conducted with the approval of the Institutional Animal Care and Use Committee (IACUC Protocol 255.15) at NYBC.

### Recombinant *Ov*-ASP-1 (rASP-1) adjuvant

The recombinant *Ov*-ASP-1 (rASP-1) protein used for the *in vivo* mouse influenza challenge model as well as for the human *in vitro* studies was expressed using T7-promoter driven pET28a expression vector (Novagen, Gibbstown, US) as described previously (17). To make sure that the residual LPS within the rASP-1 protein does not influence its adjuvanticity *in vivo* and *in vitro*, we pre-treated the protein with Polymyxin B sulfate (Sigma-Millipore) before use, and as previously described (17). Polymyxin B neutralizes the effect of LPS if present as a contaminant in recombinant proteins (20) and is also used to remove LPS from purified recombinant proteins using affinity columns (18). Briefly, rASP-1 was incubated with 30 µg/ml Polymyxin B at room temperature for 60 minutes before use. In the mouse *in vitro* experiments we used rASP-1 that was expressed using ClearColi^®^ BL21(DE3) competent cells with a modified lipopolysaccharide (LPS) (Lipid IV_A_) that does not trigger endotoxin response in human cells. Although the ClearColi expressed rASP-1 had some residual endotoxin (~ 0.0084 EU per the 1 µg of rASP-1 used per well and in each assay condition), we reasoned that there was no need to neutralize such low levels of residual endotoxin as rASP-1 did not activate TNF-α secretion *via* the MyD88-dependent pathway as expected when mouse cells are activated with endotoxin. As shown below, the ClearColi expressed rASP-1 evidently activated mouse BMDCs to secrete TNF-α, as well as IL-12p40, IP-10 and IFN-β, in a MyD88-independent manner.

### Mouse CD11c^+^ BMDC generation and activation

Bone marrows from femur and tibia of hind legs from 6-8 wks old female C57BL/6J mice were harvested, decellularized in complete R10 media [RPMI containing Glutamax and supplemented with 1X Penicillin and Streptomycin antibiotics, 1X β-mercaptoethanol (Gibco, USA), and 10% heat-inactivated (56°C for 1 hr) Fetal Bovine Serum (HyClone, USA)]. Decellularized cells were passed through a cell strainer of 70 µm size. 10 mL of cells were then cultured at ~4x10^5^/mL in 100 mm dish in the presence of 20 ng/mL of rmGM-CSF (R&D systems, Minneapolis, USA). On Day 3, 20 ng/mL of rmGM-CSF in 10 mL R10 media was added to each dish. On Day 6, suspended cells were collected in 50 mL tube, plates were rinsed twice with 5 mL of PBS (Gibco-Thermo Fischer Scientific, Massachusetts, USA) and the detached loosely adherent cells were added to the 50 mL tube. The cells were spun at 350 g for 6 minutes, resuspended in fresh R10 media and counted. The cells were then enriched for CD11c^+^ DC cells using CD11c^+^ microbeads by positive selection using MACS LS columns as per manufacturer’s protocol (Milteni Biotech, Bergisch Gladbach, Germany). Purified CD11c+ BMDCs were plated in 96-well round bottomed plates (2.5 x 10^5^ per well) and stimulated for 24 hr with 5 μg/mL of rASP-1, 1 µg/mL of monophosphoryl lipid A (MPLA, *In vivo*gen, California, USA) or left unstimulated (media). In parallel, purified CD11c^+^ BMDCs were treated with 50 µM of MyD88 inhibitor (here after MyD88X) peptide set (Novus Biologicals, Colorado, USA), 5 µM BX795 (a TBK1/IKKε inhibitor) (*In vivo*gen, California, USA) 6 hrs prior to activation with rASP-1 or MPLA, a TRIF-biased TLR4 agonist (21). The TBK1/IKKε is found downstream of TLR-mediated TRIF, dsDNA-mediated STING, dsRNA-mediated RIG-I, and MDA-IPS-1 pathways. Since our earlier studies have shown that the rASP-1-stimulated human MoDCs had multiple upregulated genes associated with the MyD88-independent TLR4 pathway, we focused on the ability of BX795 to specifically inhibit the TLR-mediated TRIF pathway. Accordingly, MPLA pre-treated with BX795 was used as the positive control for its inhibitory activity of the TLR-mediated TRIF pathway. Hence, we refer to BX795 as a TRIF inhibitor throughout this manuscript. At the end of the stimulation with the adjuvants in the presence or absence of the inhibitors, the cells within the plates were spun at 350 g for 6 minutes, culture supernatants were collected and stored at -80°C until analyzed for cytokine secretion. The cells were analyzed for the expression of DC activation markers using flow cytometry analysis. We generated BMDCs from bone marrows from four WT mice and two IFNAR^-/-^ mice on the same day and performed all the experiments with WT BMDCs and the adjuvants with and without the MyD88 and TRIF inhibitors, as well as a control for BMDCs from IFNAR^-/-^ mice experiments on the same day.

### Flow cytometry analysis of mouse activated CD11c^+^ BMDCs

Activated CD11c^+^ BMDCs were stained with a combination of fluorescently labeled antibodies specific to CD45 (I3/2.3), CD11c (N418), CD40 (3/23), CD80 (16-10A1) and CD86 (GL-1) in cell stain buffer (Biolegend, California, USA) for 20 min in the dark at 4°C. The stained cells were then washed and resuspended in the stain buffer for the analysis. The data were acquired using BD LSRFortessa cell analyzer (BD Bioscience) and then analyzed using FlowJo software (BD Biosciences).

### Cytokine analysis in activated CD11c^+^ BMDCs culture supernatants

Culture supernatant from activated CD11c^+^ BMDCs were analyzed for IL-12p40, TNF-α and IP-10 using the Luminex platform and according to manufacturer’s protocol (Millipore Sigma). IFN-β was analyzed by ELISA according to manufacturer’s protocol (PBL Assay Science)

### Naïve CD4^+^ T cell differentiation by activated CD11c^+^ BMDCs

Spleens of 6-8 weeks C57BL6/J female mice were harvested. Single cell suspension of splenocytes were prepared by gently dissociating the spleens with the back of a syringe plunger against a 70 µm size cell strainer. The cell suspension was washed with R10 media and then lysed with RBC lysing solution (Biolegend, California, USA) for 10 minutes in the dark followed by topping with extra R10 media. The cells were spun, washed with R10 media, re-suspended in R10 media, counted, and processed for CD4+ T cell purification using CD4^+^T cell microbeads by negative selection using LS columns as per manufacturer’s protocol (Milteni Biotech, Bergisch Gladbach, Germany). Cell purity was >98%. The CD4^+^ T cells were then labeled with CFSE (Carboxyfluorescein succinimidyl ester) -dye following manufacturer’s protocol to determine percentage of proliferating cells (Invitrogen, USA). Activated and washed CD11c^+^ BMDCs from wild type (WT) or IFNAR deficient mice (IFNAR^-/-^) were cultured in round-bottomed 96-well plates with allogeneic naive WT CD4^+^ T cells (1 x 10^5^ cells/well) in R10 media at 1:5 CD11c+ BMDC: naïve CD4^+^ T cell ratio for 4 days. CD4^+^ T cells stimulated with αCD3+ αCD28 (Biolegend, USA) served as the positive control. Flat bottomed plates were coated with 5 µg/mL of αCD3 in PBS (Gibco, USA) and after 2 hr incubation at 37°C the plates were washed with PBS twice before WT CD4^+^ T cells (1 x 10^5^ cells/well) in R10 media were added, followed by the addition of 2.5 µg/ml of αCD28 and incubated for 4 days.

To phenotype the differentiated CD4^+^ T cells the primed CD4^+^ T cells were restimulated on day 4 with PMA (500 ng/mL) and ionomycin (1 µg/mL) (Millipore Sigma, USA) for 6 hr in the presence of Brefeldin A (Invitrogen, USA) in the last 5 hr. Cells were first stained with CD4-PECy7 antibodies to determine percentage of proliferating CD4^+^ T cells. To determine intracellular cytokines (ICS), fixed and permeabilized cells were stained with fluorochromated antibodies to IFN-Ɣ (XMG1.2), IL-21 (mhalx21), IL-17A (TC11-18H10.1) and analyzed by flow cytometry, BD Fortessa (BD Biosciences, USA).

### Mice

C57BL/6 WT 6–8 weeks old female mice, TLR4^−/−^ (B6.B10ScN-Tlr4lps-del/JthJ), TRIF^−/−^ (C57BL/6J-Ticam1Lps2/J), IFNAR^−/−^ (B6.129S2-Ifnar1tm1Agt/Mmjax) and AID^-/-^ (B6.129P2-Aicda<tm1Mak>/J) mice were purchased from Jackson Laboratory (Bar Harbor, Maine).

### Immunization with rASP-1-adjuvanted IIV3 vaccine

The trivalent inactivated influenza vaccine (IIV3) used in this study was the seasonal Novartis 2015–16 vaccine. The vaccine contains 15 µg of hemagglutinin (HA) from each of the following three viruses in 0.5 mL volume: A/California/7/2009 NYMC X-181 (H1N1); A/Victoria/210/2009 NYMC X-187 (H3N2); and B/Brisbane/60/2008. Mice were immunized intramuscularly (i.m.) with 2.5 µL IIV3 containing 0.225 µg total HA or 0.075 µg H1N1 HA) or with 2.5 µL IIV3 formulated with 12.5 µg of rASP-1 pre-treated with Polymyxin B for 60 min. Control mice were injected with PBS containing 0.1% SDS admixed with a similar final amount of Polymyxin B; the buffer solution of rASP-1 is referred to as naïve hereafter. Twenty-three days post immunization mice were challenged intranasally (i.n.). with the H1N1/Cal/09 influenza virus.

### Protection against influenza virus

Influenza A/California/7/2009 (H1N12009) virus strain was propagated in specific pathogen-free fertile chicken eggs and purified as described before (22). Groups of 3–5 mice were challenged i.n. with 35 µL of 7500TCID50 of H1N1 Cal/09 influenza virus 23 days post the primary immunization. Mice were anesthetized intraperitoneally with 2 mg ketamine and 0.15 mg xylazine per 20 g of body mass before the inoculation with influenza virus. The vaccine-induced protective efficacy was measured by following the weight of the mice and their survival over 15 days post challenge. Infected mice were weighed daily and mice that lost >25% of their initial body weight pre-challenge were sacrificed according to IACUC guidelines. The weight represented in the figures thereafter is only from surviving mice in the various groups on the day of recording.

### RNA-seq library generation and pre-processing

Total RNA was isolated from activated cDCs using the Qiagen RNeasy Mini Kit (Qiagen) or Arcturus PicoPure (Life Technologies) kits following manufacturer’s protocols. During RNA isolation, DNase treatment was additionally performed using the RNase-free DNase set (Qiagen). RNA quality was checked using an Agilent 2100 Expert bioanalyzer (Agilent Technologies). RNA quality was reported as a score from 1 to 10, samples falling below threshold of 8.0 were omitted from the study. RNA-seq libraries were prepared with KAPA mRNA HyperPrep kit (Roche) according to manufacturer’s instruction. Poly(A) mRNA was isolated from 60 ng total RNA using oligo(dT) magnetic beads and then fragmented at 85°C for 6 minutes, targeting 250-300 bp-range fragments. Fragmented RNA was reverse transcribed by incubating it for 10 minutes at 25°C followed by 15 minutes at 42°C and finally inactivated for 15 minutes at 70°C. This was followed by second strand synthesis at 16°C for 60 minutes. cDNA was purified using AMPure XP beads (Beckman-Coulter), then A-tailed and ligated with Illumina unique adapters (Illumina). Adapter-ligated cDNA was purified using AMPure XP beads and amplified by 10 cycles of PCR. The final libraries were cleaned up using AMPure XP beads and quantified using real-time qPCR (Thermo Fisher). The libraries were sequenced on Illumina HiSeq4000 platform generating paired end reads of 75bp.

### Differential gene expression and pathway analysis

Differential analysis of transcript expression was carried out using the R/Bioconductor edgeR package (https://bioconductor.org/packages/release/bioc/html/edgeR.html). Statistical significance was set to 5% false discovery rate (FDR; q-value <0.05). Differentially expressed genes induced by stimulation of human cDCs with rASP-1 were imported into the Ingenuity Pathways Analysis (IPA) software (Ingenuity Systems; www.ingenuity.com) and were subjected to canonical pathway analysis as well as upstream regulator analysis associated with the identified gene expression profile. Only the significantly impacted pathways (p-value ≤ 0.01), calculated with the right-tailed Fisher’s exact test, were selected.

### Human cDC isolation and *in vitro* activation

Human PBMCs, used as source of cDCs, were isolated from leukopaks obtained from healthy adult volunteers (New York Blood Center) by Ficoll gradient centrifugation. Isolation of untouched cDCs was performed using immunomagnetic negative selection using a cocktail of antibodies that bind to cells that are negative of myeloid DCs (EasySep human myeloid DC enrichment kit, StemCell Technologies). All cells were cultured in RPMI 1640 medium supplemented with 1% L-glutamine, 1% penicillin/streptomycin, 1% sodium pyruvate, 1% non-essential amino acids, and 10% heat-inactivated fetal bovine serum (RPMI complete medium). cDCs (0.5 x 10^6^ per condition) were incubated in 24-well plates for 24 hours in the absence or presence of either rASP-1 (5 µg/mL) or MPLA (1 µg/mL) (positive control). After stimulation, activated cDCs were harvested and washed. Magnitude of cDCs activation was determined by flow cytometry analysis of Class II and co-stimulator expression after individual staining with antibodies specific to HLA-DR (LN3), CD40 (5C3), CD80 (2D10), CD83 (HB15e), and CD86 (FUN-1), relative to appropriate isotype-matched control staining. Data were acquired using a Fortessa X-20 (BD Biosciences) and analyzed with FlowJo software (BD Biosciences)

### CD4^+^ T cell differentiation by activated allogeneic human cDCs

Naïve CD4^+^ T cells were purified from PBMCs using immunomagnetic negative selection kit (EasySep Human Naïve CD4^+^ T cell separation kit II, StemCell Technologies); cell purity was >98%. For assessment of cDC stimulatory capacity, activated cDCs were cultured in round-bottomed 96-well plates for 7 days with allogeneic naive CD4^+^ T cells (5 x 10^4^ cells/well) in RPMI complete medium at 1:80 cDC:naïve CD4^+^ T cell ratio. For the phenotype analysis of the differentiated CD4^+^ T cells, recovered cells were stained with fluorochromated antibodies specific to CD3 (UCHT1), CD4 (OKT4), CD45RA (3H4), CXCR5 (JD252D4), CXCR3 (G025H7), CCR6 (11A9), ICOS (C398.4A), and PD-1 (MIH-4). Dead cells were excluded from the analysis by labeling the cells with LIVE/DEAD fixable Aqua. For the analysis of intracytoplasmic cytokine expression, cDC-primed and differentiated CD4^+^ T cells were restimulated on day 7 with PMA (25 ng/mL) and ionomycin (1 µg/mL) for 6 hr and in the presence GolgiPlug (BD) for the last 4 hr. Fixed and permeabilized cells were stained with fluorochromated antibodies to IL-21 (3A3-N2), IL-17A (BL168), IL-13 (JES10-5A2), and IFN-Ɣ (B27) and analyzed by flow cytometry. Dead cells were excluded from the analysis by labeling the cells with LIVE/DEAD fixable Aqua prior to fixation/permeabilization.

### Statistical analysis

Mouse CD11c^+^ BMDC data were expressed as mean ± SEM. Unpaired, two-tailed Student’s t-test (Man-Whittney) was used to determine significance between each two treatment groups in WT mice. Paired, Wilcoxon-signed rank test was used to determine the significance between adjuvant treated cells in the presence and absence of inhibitors or in adjuvant treated cells between WT and IFNAR^-/-^ mice. P< 0.05 was considered statistically significant. Unpaired, two-tailed Student’s t-test was used to determine significance between each two treatment groups involving human cDCs. Human cDC data were expressed as mean ± SD. P< 0.05 was considered statistically significant.

## Results

### rASP-1 activates mouse CD11c^+^ BMDCs *in vitro* in a TRIF-dependent manner

**Figure 1:**
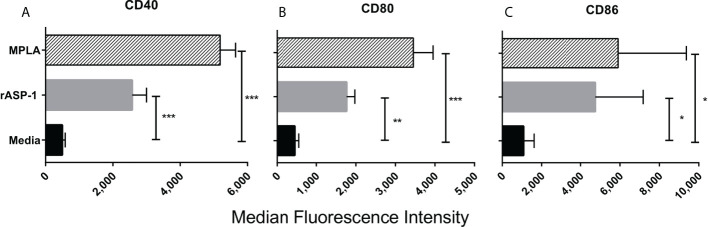
rASP-1-activated murine CD11c^+^ BMDCs express significantly higher levels of co-stimulatory molecules. Murine CD11c^+^ BMDCs were stimulated for 24 hr with rASP-1 (5 μg/ml) or MPLA (1 μg/ml). Unstimulated media served as the control. Cells were harvested and the expression of **(A)** CD40, **(B)** CD80, and **(C)** CD86 was quantified by median fluorescence intensity as measured by FACS. Data represents median fluorescent intensity ± SD of three individual experiments. Each stimulation was done in duplicate wells. Statistical analysis was performed using Mann Whitney U test. * P <0.05, ** P <0.01 and *** P <0.001.

**Figure 2:**
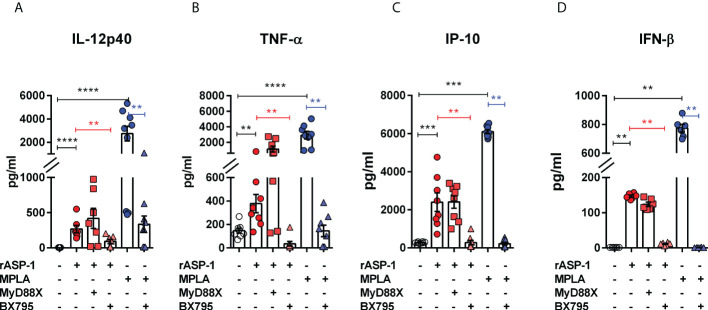
rASP-1-activated murine CD11c^+^ BMDCs secrete significantly increased levels of cytokines and IP-10 that are associated with Th1 responses and IFN-β in a TRIF-dependent manner. Murine CD11c^+^ BMDCs were stimulated for 24 hr with rASP-1 (5 µg/ml) or MPLA (1 μg/ml) in the presence or absence of MyD88X (50 μM) or BX795 (5 μM) wherever indicated. Unstimulated media and MPLA served as negative and positive controls, respectively. Culture supernatants were analyzed for **(A)** IL-12p40, **(B)** TNF-α, **(C)** IP-10, and **(D)** IFN-b. Data represents mean ± SEM of 2-4 individual experiments. Each individual experiment had at least two technical replicates. MyD88X, MyD88 inhibitor; BX795, TRIF inhibitor. Statistical analysis was performed using Mann Whitney U test for unpaired ‘t test’ between and Wilcoxon-signed rank test for paired t test between presence and absence of inhibitors. ** P < 0.01, *** P < 0.001, **** P < 0.0001.

The effect of rASP-1 on mouse DCs was tested *in vitro* using CD11c^+^ BMDCs generated by culturing bone marrow cells with GM-CSF for 6 days. Activation was assessed after 24hr. ASP-1 induced a significant increase of the expression of CD40, CD80 and CD86 (**Figure 1**) and rASP-1 increased the secretion of IL-12p40, TNF-α and IP-10 (interferon γ-induced protein 10 kDa) (**Figures 2A–C**). The first two cytokines participate in the Th1 polarization of CD4^+^ T cells (23, 24), whereas IP-10 is a known chemoattract of innate cells and activated T cells (25) and promotes Th1 responses (26, 27). Notably, pretreatment of BMDCs with the BX795 (referred to as TRIF inhibitor thereof) significantly reduced the rASP-1-induced production of IL-12p40, TNF-α and IP-10. In contrast, pretreatment with the MyD88 inhibitor (MyD88X) (**Figures 2A–C**) had no effects. Moreover, rASP-1 BMDCs secreted IFN-β (**Figure 2D**), but not IFN-α (data not shown). Pretreatment of the BMDCs with the TRIF inhibitor but not with the MyD88 inhibitor significantly reduced IFN-β secretion (**Figure 2D**). As expected, the TRIF inhibitor reduced the MPLA (a TRIF-dependent TLR4 ligand) induced production of IL-12p40, TNF-α, IP-10 and IFN-β by BMDCs (**Figures 2A–D**). In addition, BMDCs were pre-treated with the MyD88 inhibitor before activation with Pam3CSK4 (a MyD88-dependent TLR2 ligand) as a control for MyD88-dependent activity. As expected, the MyD88 inhibitor reduced the Pam3CSK4 induced production of IL-12p40 and TNF-α (**Figure S1**).

### rASP-1-activated BMDCs promote the differentiation of naïve CD4^+^ T cells into Th1, Tfh-like and Tfh1 cells

**Figure 3:**
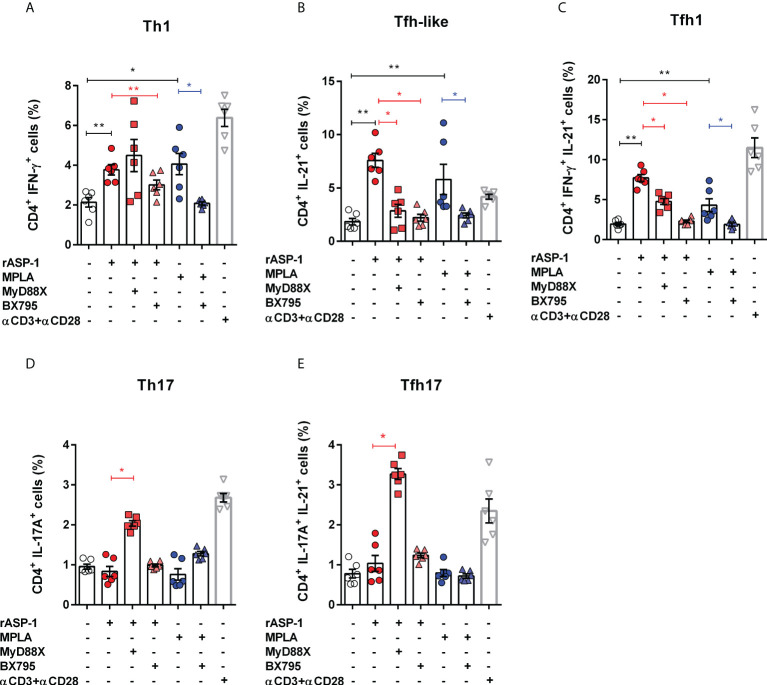
rASP-1-activated murine BMDCs stimulates differentiation of naïve CD4^+^ T cells into Th1, Tfh-like and Tfh1cells in TRIF-dependent manner. Activated BMDCs were co-cultured with naïve CD4^+^ T cells for 4 days in 1:5 ratio. aCD3^+^ aCD28 stimulated naïve CD4^+^ T cells alone served as the positive control. Percentage of differentiated CD 4^+^ T cells that express cytokines associated with **(A)** Th1 cells (IFNg ^+^), **(B)** Tfh-like cells (IL-21^+^), **(C)** Tfh1co-expressing IFN-g and IL-21 (IFNg^+^IL-21^+^), **(D)** Th17 cells (IL-17A^+^), and **(E)** Tfh17cells co-expressing IL-17A and IL-21 (IL-17A^+^IL-21^+^) as determined by flow cytometry analysis. Data represents mean + SEM of two individual experiments. Each individual experiment had three technical replicates. MyD88X: MyD88 inhibitor, BX795: TRIF inhibitor. Statistical analysis was performed using Mann Whitney U test for unpaired ‘t test’ between and Wilcoxon-signed rank test for paired t test between presence and absence of inhibitors. * P < 0.05, ** P < 0.01.

We tested the ability of the rASP-1-activated BMDCs to induce T cell differentiation by culturing them with allogenic naïve mouse CD4^+^ T cells for 4 days and assessed their intracellular cytokine expression. Therefore, based on their intracellular cytokine expression, we named the CD4^+^ T cells expressing IL-21 as “Tfh-like” cells, cells that co-express both IFN-γ and IL-21 as “Tfh1” cells and cells that co-express both IL-17A and IL-21 as “Tfh17” cells (28, 29). The rASP-1-activated BMDCs induced the expression of IFN-γ^+^ (Th1), IL-21^+^ (Tfh-like) or IFN-γ^+^IL-21^+^ (Tfh1) but not IL-17A^+^ (Th17) in the CD4^+^ T differentiated cells (**Figures 3A–E**, **Figure S2**). Of note, the differentiated mouse Tfh-like cells (IL-21^+^) did not express the CXCR5^+^PD-1^+^ markers of Tfh cells.

BMDCs activated with rASP-1 in the presence of the TRIF inhibitor (BX795), but not the MyD88 inhibitor (MyD88X), failed to promote differentiation of naïve CD4^+^T cells into Th1 cells (IFNγ^+^) (**Figure 2A**). Surprisingly, BMDCs activated with rASP-1, in the presence of TRIF or MyD88 inhibitors, enhanced the differentiation of CD4^+^ T cells into IL-17^+^ (Th17) cells and IL-17^+^IL-21^+^ (Tfh17) cells (**Figures 3D, E**) while inhibiting the differentiation of CD4^+^ T cells into IL-21^+^IFNγ^-^ (Tfh-like) and IL-21^+^IFNγ^+^ T cells (Tfh1) (**Figures 3B, C**).

The MPLA-activated BMDCs promoted the differentiation of naïve CD4^+^ T cells into Th1 cells (IFN-γ^+^) and Tfh-like cells (**Figures 3A–C**) but not into Th17 cells (IL-17A^+^) (**Figures 3D, E**). Whereas the PAM3CSK4-activated BMDCs promoted the differentiation of naïve CD4^+^ T cells into Th1, Th17, and IL-17A^+^IL-21^+^ cells, but not into Tfh-like or Tfh1 cells (**Figure S3**).

### Differentiation of naïve CD4^+^ T cells by rASP-1-activated BMDCs is type I IFN receptor-dependent

**Figure 4:**
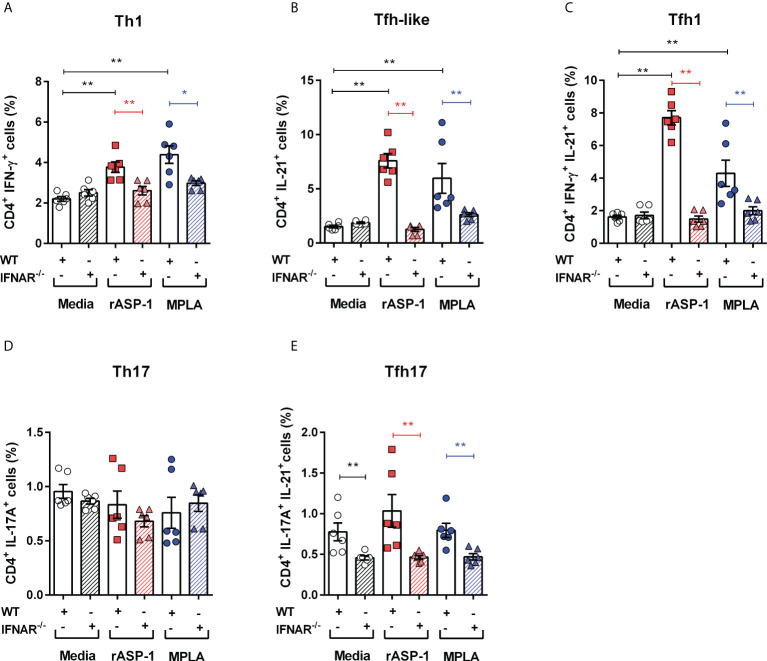
rASP-1-activated murine BMDCs differentiated naïve CD4^+^ T cells to Th1, Tfh-like and Tfh1 cells was type I IFN receptor dependent. Activated BMDCs from wild type (WT) mice and type I IFN receptor deficient mice (IFNAR-/-) were co-cultured with naïve CD4^+^ T cells from WT mice for 4 days. Percentage of differentiated T cells that express cytokines associated with **(A)** Th1 cells (IFN-g ^+^), **(B)** Tfh-like cells (IL-21^+^), **(C)** Tfh1cells co-expressing IFN-g and IL-21 (IFN-g ^+^IL-21^+^), **(D)** Th17 cells expressing IL-17A, and **(E)** Tfh17cells expressing both IL-17A and IL-21 in WT and IFNAR-/- mice as determined by flow cytometry analysis. Data represents mean + SEM of two individual experiments. Data of WT mice were same as seen in **Figure 3**. Each individual experiment had three technical replicates. WT: wild type C57BL/6J mice, IFNAR-/-: IFNAR-deficient mice of B6 background. Statistical analysis was performed using Mann Whitney U test for unpaired ‘t test’. * P <0.05, ** P <0.01.

We next examined the role of type I IFN in the differentiation of naive CD4^+^ T cells by rASP-1-activated BMDCs. Thus, rASP-1-activated BMDCs from IFNAR^-/-^ mice failed to promote the differentiation of naïve CD4^+^ T cells into Th1 (IFN-γ^+^), Tfh-like (IL-21^+^) cells or into Tfh1 (IFN-γ^+^IL-21^+^) cells (**Figures 4A–E**, **Figure S4**). Likewise, MPLA-activated BMDCs from IFNAR^-/-^ mice were poor inducers of naïve CD4^+^ T cell differentiation. rASP-1-activated BMDCs from WT or IFNAR^-/-^ mice did not promote the differentiation of Th17 (IL-17A^+^) or Tfh17 (IL-17A^+^ IL-21^+^) cells (**Figures 3D, E**). The CD4^+^ T cells expressing both IL-17A and IL-21 were significantly reduced in all conditions including the unstimulated IFNAR^-/-^ BMDCs (**Figure 4E**).

### rASP-1 activates multiple immune-related signaling pathways in human cDCs

**Figure 5:**
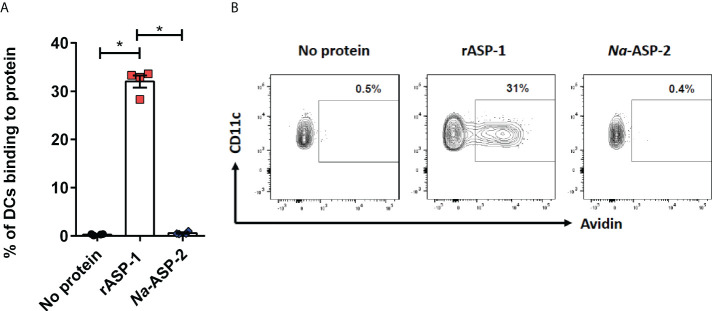
rASP-1 binds to human CD11c^+^ cDC cells. PBMCs (1 x 10^6^) were incubated in the presence or absence of 5 µg biotinylated rASP-1 or *Na*-ASP-2, a homologue of rASP-1 used as the negative control protein. Specific binding was detected with fluorochromated avidin and flowcytometry. **(A)** the frequency of positive binding within the gated CD11c^+^ DCs. Representative FACS plot of binding profiles of CD11c+ in the **(B)** absence or presence of biotinylated rASP-1 or *Na*-ASP-2. Data in **(B)** represents mean ± SEM of binding outcomes in two independent experiments using PBMCs from four different donors. Statistical analysis was performed using Mann Whitney U test. * P < 0.05.

**Figure 6:**
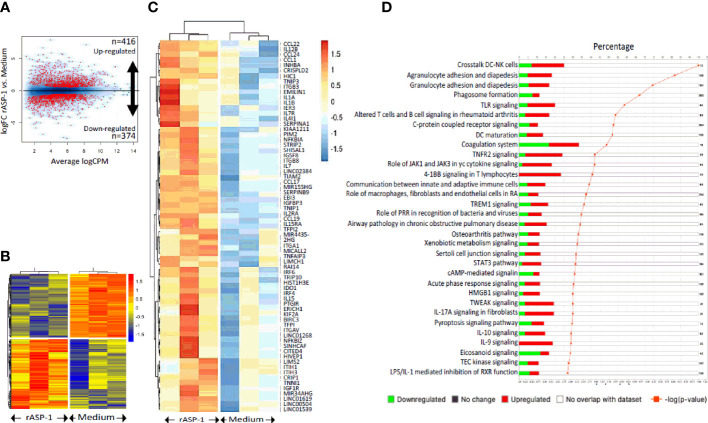
rASP-1 is a potent transcriptional activator of the immune-related genes. cDCs isolated from three different donors were exposed to medium alone or to rASP-1. At 24h post exposure, RNA was isolated and gene expression levels was measured by bulk RNA-seq. **(A)** Volcano plot showing the differentially expressed genes (DEGs) upon rASP-1- stimulation. 416 and 374 genes were found to be up- and down-regulated respectively. **(B)** Heat map representing the DEG (n=790). Each column represents a sample and each row a gene. **(C)** Heatmap representing the scaled expression values of the top 69 up-regulated immune-related genes. **(D)** Ingenuity Pathway Analysis (IPA) annotation of DEGs. Each bar represents a pathway. Red and green colors represent the percentage of up- and down regulated genes respectively. Only significant enrichments are displayed (p-value ≤ 0.01, orange line). The number of genes encompassed in each pathway is shown on the right.

Since differences in the type I IFN-mediated regulation of the Th1 responses between mice and humans have been reported (30), we analyzed the response of human blood cDCs to rASP-1 activation. First, data illustrated in **Figure 5** show that rASP-1 binds specifically to ~ 30% of blood CD11c^+^ human cDCs. For comparison, *Na*-ASP-2 (negative control protein), a homologue of the *Ov*-ASP-1 protein expressed by another helminth, *Necator americanus* also known as hookworm, did not bind to CD11c+ cDCs. The gating strategy of CD11c^+^ DCs is shown in **Figure S5**. We then analyzed the transcriptome of cDCs activated with rASP-1 (n=3). Principal component analysis (PCA) based on total gene detected (**Figure S6A**) or a set of gene-sets, including interferon (**Figure S6B**) and cytokine related genes (**Figure S6C**), clearly distinguished rASP-1-activated cDCs from the controls. Differential analysis revealed that stimulation with rASP-1 upregulated the expression of 416 and downregulated that of 374 genes (**Figures 6A, B**). The most significant upregulated gens were *CCL22* and *CCL19* that are associated with chemotaxis to T cells and T helper cell differentiation, respectively. Immune-related genes were also highly represented in the top 69 differentially up-regulated genes and included genes such as *IL12B*, *IL1β* and *IL1α* (**Figure 6C**). Moreover, Ingenuity Pathway Analysis (IPA), revealed that many of the upregulated genes were those associated with “TLR signaling”, and “DC maturation” (**Figure 6D**).

Analysis of the TLR signaling pathways (**Figure S7**) revealed the upregulation of *TRAF* and *TCAM1*, known activators of the NFkB and IRF3/7 transcription factors. Notably, activation of NFkB leads to induction of pro-inflammatory cytokines while activation of IRF3/7 leads to the induction of type I IFNs (IFN-α and -β) and the chemokine CCL5 (**Figure S7B**). Analyses of the “DC maturation” pathway confirmed the upregulation of *CD40*, *CD80*, *IL12* and *IL15*, whereas the upregulation of *IL15r* and *ICAM1* suggest a potential crosstalk between matured DCs and NK cells (**Figure S8**). Several signal transducer and activator of transcription (STAT) factors associated with T cell proliferation and differentiation were also upregulated (**Figure S9A**). IL-21 signaling through STAT3 phosphorylation is known to drive Tfh responses (**Figures S9B, C**), as observed *in vitro*. It also must be noted that the results from IPA (**Figures S7**–**S9**) are used as a predictive tool (and not definitive) for hypothesizing potential mechanism(s) of action of rASP-1.

Further analysis of the differentially expressed genes (DEGs) using IPA allowed us to identify many potential upstream regulators in the rASP-1-activated cDCs (**Table 1**) having activation Z-score values above 1.8. Collectively, these upstream regulators were mostly associated with innate immune response including members of the TLR family and the Th1 cytokine, TNF-α (**Table 1**). Of note, the most upregulated gene is OSCAR, an FcRɣ-associated receptor involved in antigen presentation and activation of human DCs, that plays a role in the initiation the adaptive immune response (31).

**Table 1:** Top 21 differentially expressed genes in rASP-1-activated human cDCs and their associated targets in innate cell pathways.

Upstream Regulator	Activation z-score	p-value of overlap	Target Molecules in Dataset
OSCAR	3.464	2.77E-08	CCL24, CCR2, CD40, CD83, CXCL1, CXCL2, CXCL8, IL12B, IL1A, MAP3K8, MMP7, TLR7
TLR7	2.953	3.86E-06	CCR7, CD1D, CD274, CD40, CD80, CD83, CXCL13, CXCL8, FCMR, IL12B, IL1B, IL4I1
RNASE2	2.608	1.71E-04	CCL1, CCL22, CCL24, CSF1, IL12B, IL2RA, IL7
CSF2	2.574	4.72E-04	CD40, CD80, CXCL8, IL12B, IL1B, IRF4, LAMP3, TLR5
RNASE1	2.449	7.22E-04	CCL1, CCL22, CCL24, IL12B, IL2RA, IL7
TNF	2.407	5.66E-03	CD274, CD40, CD80, CD83, IL1B, LAMP3
SELPLG	2.333	9.11E-06	BCL2A1, CDKN1A, CXCL2, CXCL8, HCAR3, IDO1, IL1B, IL1R2, SERPINB9
TSLP	2.224	3.15E-05	CCL17, CD40, CD80, CD83, IL12B
FCGR2A	2.200	3.67E-04	CCL1, CCL22, CXCL8, IL1B, PECAM1
TLR2	2.189	1.37E-04	CCR2, CD80, CXCL8, IL12B, IL15, IL15RA, IL1B, SLC7A11
PF4	2.038	5.55E-05	CCL22, CD83, CXCL8, FN1, IL12B, IL1A, IL1B, LY75
TLR8	1.998	1.79E-03	CCR7, CD40, CD83, IL12B
S100A8	1.969	3.84E-03	CD80, CXCL8, IL1A, IL1B
CD40	1.961	2.52E-05	CD40, CD80, CD83, IL12B, IL1A, IL1B, SAMSN1
TLR4	1.882	1.99E-05	CD80, CXCL8, IDH2, IL12B, IL15, IL1B, NFKBIA, RELB, SLC7A11, TSPAN33
IL17A	1.671	5.19E-06	BCL2A1, CCL22, CD274, CD40, CD83, CXCL1, IL1A
RELA	1.414	5.19E-06	CCL19, CD274, CXCL8, IL12B, IL1A, IL1B, NFKBIA
IL4	0.506	3.49E-03	CCL17, CD80, CD83, CYSLTR1, IRF4, PDE4B, TNFRSF4
TSC22D3	0.083	1.79E-03	CD274, CD80, CD83, CXCL8
IL2	-0.063	1.10E-02	IL12B, IL18BP, IL7R, PDE4B, RFTN1
IL13	-0.401	6.41E-11	ADA, ADAMDEC1, ARNTL2, CCL22, CD101, CD36, CD52, CHN2, CHST2, CYSLTR1, F13A1, FABP4, G0S2, GPNMB, HSD11B1, IL1R2, LIPA, LTA4H, MAF, MAOA, MS4A4A, MSMO1, PPARG, RFTN1, SOCS1, TGM2, THBS1, WNT5A
TREM1	-0.555	6.59E-15	ACKR3, ADORA2B, CCL17, CCR7, CD274, CD83, CFB, CSF1, CXCL1, CXCL2, CXCL8, DUSP14, E2F7, EBI3, ELOVL7, GADD45B, HS3ST3B1, IDO1, IL12B, IL15RA, IL1B, INHBA, LAMP3, LY9, MAFF, MAP3K8, MCOLN2, NEDD4L, PIM2, PPARG, RGS1, SLAMF7, TFPI2, THBS1, TNIP3, WNT5A
ILF3	-0.853	6.60E-03	CCL22, CD300A, CD40, CD9, CXCL1, CXCL8, IL1B, PPARG, TRAF1
IL10	-1.191	2.94E-06	BCL2A1, CD274, CD40, CD80, CD83, FCGR1A, IL12B, IL1B, LAMP3, PDCD1LG2, PDE4B, SOCS3
BTK	-2.433	4.72E-03	CCR7, CD274, CD40, CXCL8, IL4I1, IRF4

### rASP-1-activated human cDCs promote differentiation of naïve CD4^+^ T cells into Th1- and Tfh-like cells

**Figure 7:**
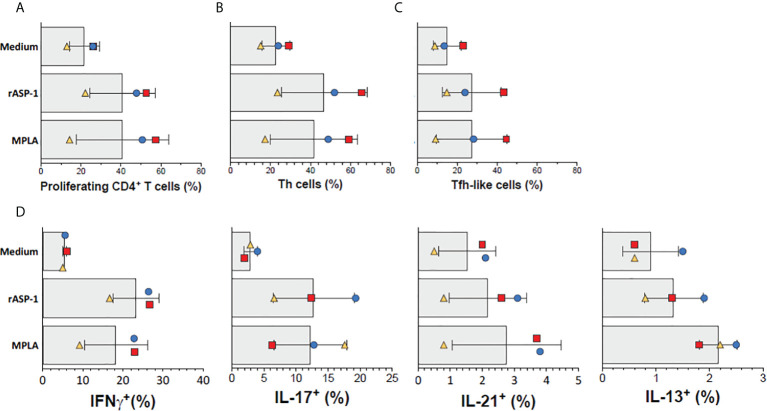
rASP-1-activated cDCs stimulate proliferation and differentiation of naïve human CD4^+^ T cells into Th- and Tfh-like cells. cDC-mediated proliferation and differentiation of naïve CD4^+^ T cells into Th- (CD4^+^CD45RA^-^CXCR5^-^) and Tfh-like (CD4^+^CD45RA^-^CXCR5^+^) cells in three independent activated cDC samples as analyzed by flow cytometry. **(A)** Frequencies of proliferating CD4^+^T cells (gated viable CD4^+^FSC^hi^ cells). **(B)** Frequencies of Th cells, **(C)** Frequencies of Tfh-like cells, **(D)** Frequencies of IFN-γ^+^ (Th1), IL-17^+^ (Th17), IL-21^+^ (Th1) and IL-13 (Th2) intracellularly expressing cells. Data shown are median frequencies ± SD from three different donors.

Finally, we tested the ability of rASP-1-activated human cDCs to promote the differentiation of allogeneic human naïve CD4^+^ T cells. Thus, rASP-1-activated cDCs, when compared to unstimulated cDCs, increased both the proliferation of naïve allogenic human CD4^+^ T cells (**Figure 7A**), and their differentiation into Th cells (CD4^+^CD45RA^-^CXCR5^-^) and Tfh-like cells (CD4^+^CD45RA^-^CXCR5^+^) (**Figures 7B, C**, **Figure S10A**). rASP-1-activated cDCs also promoted expression of intracellular IL-21 (Tfh-like), IFN-γ (Th1) and IL-17A (Th17) cytokines but not IL-13 (Th2) within the differentiated CD4^+^ T cells (**Figure 7D**, **Figure S10B**).

### Protection induced by the rASP-1-adjuvanted IIV3 vaccine is TRIF-, IFNAR- and antibody-dependent

**Figure 8:**
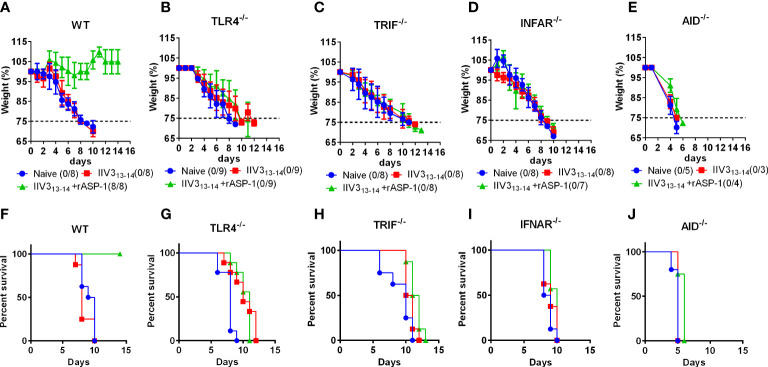
rASP-1-adjuvanted IIV3 vaccine protects mice from H1N1 challenge in a TLR4/TRIF/IFNAR and AID-dependent pathways. Mice were immunized i.m. once with IIV3 or rASP-1-adjuvanted IIV3 vaccine (IIV3 +rASP-1). Three weeks post immunization, mice were challenged i.n. with 7500TCID_50_ of H1N1. Weight of mice was recorded over a period of 14 days. Mice that have lost more than 25% of their initial body weight were euthanized. rASP-1-adjuvanted IIV3 vaccine protected **(A, F)** WT mice but not **(B, G)** TRIF^-/-^, **(C, H)** TLR4^-/-^, **(D, I)** IFNAR^-/-^ and **(E, J)** AID^-/-^ mice from H1N1 challenge. **(A–E)** represents weight loss chart over a period of 14 days and **(F, G)** indicates the percentage of survival at the end of 14 days post H1N1 challenge. Survival is indicated as those that do not lose 25% of their initial body weight. Data in WT, TRIF^-/-^, TLR4^-/-^, IFNAR^-/-^ mice experiments represent 4-5 mice per immunization group from two individual experiments. While data in AID^-/-^ mice experiment represents 3-5 mice per group from one individual experiment.

Previously, we have shown that rASP-1-adjuvanted IIV3 vaccine induced protection against H1N1 challenge was CD4^+^ T cell-dependent (17). To further determine the essential pathways in rASP-1 induced crosstalk between innate and adaptive immunity *in vivo*, mice deficient of either TRIF (TRIF^-/-^), type-I IFN receptor (IFNAR^-/-^) or TLR4 (TLR4^-/-^) were immunized i.m. with IIV3 or with rASP-1-adjuvanted IIV3 vaccine. While 100% of rASP-1-adjuvanted IIV3 immunized WT mice survived the H1N1 i.n. viral challenge (7500TCID_50_) (**Figures 8A, F**), the TLR4^-/-^, TRIF^-/-^, and IFNAR^-/-^ immunized mice succumbed to the infection (**Figures 8B–D, G–I**). This data indicates that rASP-1-adjuvanted IIV3 vaccine protects mice from H1N1 infection in a type I IFN-dependent manner *via* the TLR4/TRIF axis. Previously we also found that protection was independent on the presence of neutralizing antibodies (17) although the adjuvanted vaccine after prime immunization induced significantly elevated titers of anti-HA antibodies. To validate that vaccine induced antibodies were important for the protective adaptive immunity, mice deficient in IgG production (AID^-/-^) were immunized and challenged. The survival data (**Figures 8E, J**, **Figures S11D, E**) of the AID^-/-^ mice clearly confirm that IgG antibody responses were indispensable for the protection against H1N1 infection, while the IgM responses were not sufficient.

## Discussion

This study was designed to further characterize the functions and mechanisms of the protein adjuvant rASP-1 on mouse and human dendritic cells. Our previous studies, which showed that protection against influenza infection elicited by the rASP-1-adjuvanted IIV3 vaccine is independent of MyD88, led us to hypothesize that type I IFN, a critical element of anti-viral responses (32–34) has a significant role for this protection. The present study produced four key findings: 1) rASP-1 induces type I and type II IFN cytokine production, 2) The TRIF adaptor molecule and the IFNAR signaling are critical for the Th1, Tfh-like and Tfh1cell differentiation by rASP-1-activated BMDCs, 3) rASP-1-activated human cDCs promote differentiation of naïve CD4^+^ T cells into Th1 and Tfh-like cells, and 4) TLR4-TRIF axis and the IFNAR signaling are critical for the protection elicited by the rASP-1-adjuvanted IIV3 vaccine against the H1N1 infection in mice, as well as its ability to induce anti-IIV3 IgG antibody production.

Specifically, we show that rASP-1-activated mouse BMDCs secrete elevated levels of Th1-inducing cytokines (IL-12p40 and TNF-α, IP-10 (Th1-associated cytokines and chemokine) and IFN-β, in a TRIF-dependent and MyD88-independent fashion. Functional TRIF is also necessary for rASP-1 to activate mouse DCs to secrete IFN-β.

TRIF signaling was also critical for rASP-1-activated mouse BMDCs to promote Th1 (IFN-γ^+^) differentiation, and both TRIF and MyD88 were required to promote the differentiation of CD4+ T cells into Tfh-like (IL-21^+^) cells. Though Tfh cells are generally characterized by their surface expression of PD-1 and CXCR5 and secretion of IL-21, the rASP-1 induced Tfh-like and Tfh1 (IL-21^+^IFNγ^+^) cells did not express CXCR5, and were thus named Tfh-like cells as observed by others (35–37). Although rASP-1 activated BMDCs did not promote the differentiation of naïve CD4^+^ T cells into Th17 cells (IL-17A^+^), inhibiting MyD88 in rASP-1-activated BMDCs allowed them to induce the differentiation of Th17 (IL-17A^+^) and Tfh17 (IL-17A^+^ IL-21^+^) cells. We hypothesize that the observed inhibitory effect on the Tfh-like cells and Tfh1 cells may have been due to the combined effects of IL-1, IL-23 and TGF-β cytokines through the IL-1R signaling, as MyD88 can also stimulate IL-1 signaling during the initial commitment stage that is linked to IL-23 signaling, both of which are critical in Th17 differentiation (38). Hence, this indicates that functional MyD88 in rASP-1-activated BMDCs prevents the ability of DCs to induce the generation of Th17 cells pointing to a potential path for the induction of Th17 responses when the MyD88 pathway is non-functional in humans (38).

Since differential outcomes of immune responses exists between mice and human, especially with the Th1 responses involving type I IFNs (39), it was important to assess the immunopotentiating effects of rASP-1 on human cDCs, and the ability of the activated cDCs to promote CD4^+^ T cell differentiation. We focused this time on primary human cDCs (arise from a common DC progenitor cells) instead of monocytes derived DCs we studied before, which arise from common myeloid progenitor cells after stimulation with GM-CSF and IL-4, and have different phenotypic and functional attributes (40). Since the cDC population in human blood is at the most 0.01 to 0.1%, we were unable to secure more than three samples for analysis this time as not all the Leukopak donors (N = 8) had the required cDC numbers for setting up the assays with all the *in vitro* stimulation conditions. rASP-1 binds to a receptor expressed on ~30% of blood CD11c^+^ human DCs, whose nature remains to be determined. rASP-1 activate cDCs uniquely as was revealed by the upregulation of genes associated with TLR4 signaling including NFkB and IRF3/IRF7 transcription factors that are dependent on TRAF1-TICAM1 and not MyD88. This suggests that rASP-1 activates the TLR4 signaling *via* the TRIF adaptor molecule, as was observed *in vitro* with mouse BMDC. rASP-1-activated human cDCs also had upregulated genes associated with DC maturation and differentiation (CD40, CD80, CD86, IL-12, TNF-α), induction of type I IFNs (IRF3/IRF7). Aligned with our mouse BMDC data, rASP-1-activated cDCs also promoted the differentiation of naïve CD4^+^ T cells into Th1 (IFN-γ^+^), Tfh1 (IFN-γ^+^IL-21^+^) and Tfh-like cells (IL-21^+^). Notably, circulating Tfh/Tfh1 cells have been shown to correlate with the development of protective antibody responses generated by memory B cells upon seasonal influenza vaccination (41).

One of the most significantly upregulated gene is the CCR7 ligand *CCL19*, a chemokine produced only by mature DCs and known to be involved in chemotaxis of DCs and T cells, and the regulation of T cell activation (42). Notably, exposure of myeloid and plasmacytoid DCs to influenza virus stimulates production of three sequential waves of cytokines and chemokines depending on the state of DC maturation; CCL19 is secreted only when DCs reached the lymphoid organs (43, 44). Thus, the upregulation of *CCL19* chemokine is another confirmation for the ability of rASP-1 to induce maturation of cDCs also through this chemokine. Additionally, the CCL19-CCR7 axis was shown to promote hepatitis virus clearance in chronic hepatitis B patients through T cell activation and identified that CD4^+^ T cells expressing CXCR5 (Tfh) and IFN-γ were positively correlated with immune activation (45), which is aligned with the observed ability of the rASP-1 activated human cDCs to induce differentiation *in vitro* of T cells into Th1 and Tfh1 cells (**Figure 7**).

Among the top genes upregulated by rASP-1 was also *CCL22*. The chemokine CCL22 binds to its receptor CCR4 that is highly expressed on several T cell subtypes as well as DCs. CCL22 mediates chemotaxis of CCR4 expressing T cells, thus promoting the migration of these cells to tissues such as lymph nodes or lungs (46). Notably, transcriptional studies in lung tissues of Estriol treated mice post influenza A infection revealed a correlation between upregulation of *CCL19* and *CCL22* genes with reduced lung inflammation and pathology due to influenza (47).

rASP-1-activated cDCs also showed increased expression of genes that are associated with the crosstalk between DCs and NK cells including *IL15*, *IL15r*, *IL12*, and type I IFNs. In this context, we had previously reported that rASP-1 enhanced recall responses to hepatitis C virus and tetanus toxoid that was dependent of IFN-γ secreting CD56^+^ NK cells (15). The potential crosstalk between rASP-1 activated DCs and NK cells revealed in the current transcriptome data will require further exploration to determine whether this is a unique rASP-1 mediated immunopotentiating activity.

In line with the ability of type I IFNs to promote DC maturation and T cell differentiation (33, 48–50), we determined that the IFNAR was critical for the rASP-1-activated BMDCs to promote differentiation of naïve CD4^+^ T cells into Th1, Tfh-like and Tfh1 cells. The indispensable roles of TRIF adaptor molecule and IFNAR for the immunopotentiating properties of rASP-1 *in vitro* were validated *in vivo* as rASP-1-adjuvanted IIV3 vaccine elicited in mice protection against a lethal H1N1 infection that was dependent on pathways involving the TLR4-TRIF-axis and IFNAR signaling (**Figures 8A–D**).

Although type I IFNs represent a family of cytokines with antiviral functions, previous studies showed that type I IFNs have critical role also in Tfh differentiation, isotype class switching and induction of enhanced humoral immunity (51, 52). AID (the activation-induced cytidine deaminase) is known to be highly expressed in germinal centers and necessary for class switch recombination (CSR) of IgM genes to other isotypes (53). Our *in vivo* data with AID deficient mice validated that IgG, but not IgM (despite elevated levels) antibody responses were indispensable in the protection against H1N1 infection (**Figures 8E, J**, **Figures S11D–E**). We also show, and had published earlier, that IgG1 or IgG2c antibody responses elicited by rASP-1-adjuvanted IIV3 are critical for protection from H1N1 infection (17, **Figures S11A–C, E**). Interestingly, rASP-1-adjuvanted IIV3 immunized mice deficient of TLR4, TRIF and IFNAR had significantly lower IgG1 and IgG2c antibody responses versus WT mice. Although there were significantly elevated levels of IgG1 responses in IIV3 immunized mice deficient of TLR4 and TRIF versus WT, there was no significant change in the IgG2c levels in IIV3 immunized deficient mice versus WT mice. It is to be noted that IgG2c antibodies have protective potential through Fc-mediated antibody functions against influenza and other viral pathogens (54). As our previous study has shown that the rASP-1-adjvanted IIV3 vaccine induced protection was independent of HA-neutralizing antibodies but dependent on CD4+ T cells (17), we posit that the rASP-1-adjuvanted IIV3 vaccine protects mice through other antibody-dependent mechanisms such as antibody-dependent cell-mediated cytotoxicity (ADCC) and/or antibody-mediated phagocytosis. Non-neutralizing antibodies were shown to play a role in protection from several viral pathogens including influenza, HIV, herpes, alphaviruses, flaviviruses, respiratory syncytial virus, and CMV (55, 56). ADCC functions through antibody binding to the Fc receptors of effector cells (57, 58). interestingly, the current human cDC transcriptome data has indicated the upregulation of genes associated with defined Fc receptors; *OSCAR* and *FCGR2A* (**Table 1**), which might support our hypothesis that ADCC might be an alternate mechanism by which the rASP-1 adjuvant exerts it potency after primary immunization when used with the influenza vaccine.

Overall, our findings revealed a critical role of the TRL4-TRIF axis and IFNAR signaling for the adjuvanticity of rASP-1. Since type I IFN signaling is critical for protection against several viral infections, rASP-1 stands to be a new addition to other known type I IFN stimulating adjuvants such as Poly I:C (TLR3 agonist), R848 (TLR7/8 agonist), CpG (TLR9 agonist) and STING agonists. Presently only the R848 and CpG adjuvanted Hepatitis B vaccines were evaluated in phase III clinical trials against viral infection (NCT00175435, NCT02117934), while a STING agonist is being tested as an adjuvant in cancer immunotherapy but not against viral infections (NCT03172936). As a new addition to the type I IFN stimulating adjuvants, and because type I IFN signaling is dysregulated in aged individuals who also might have poor immune responses (59–61), it will be of interest to investigate whether the rASP-1 adjuvant has also immunopotentiating properties in aged mice and on elderly human cells, and ultimately restore the dysregulated type I IFN signaling pathways associated with aging.

## Data availability statement

The data presented in the study are deposited in the NCBI/GEO repository, accession number GSE196923.

## Ethics statement

Ethical review and approval was not required for the study on human participants in accordance with the local legislation and institutional requirements. The animal study was reviewed and approved by the Institutional Animal Care and Use Committee (IACUC Protocol 255.15) at NYBC.

## Author contributions

Conceptualization: PG, SL, RM, and JB; methodology: PG, RM, and DN-B; software: PG, RM, and DN-B; validation: RM, SL, and JB; formal analysis: PG, RM, and DN-B; investigation: SL and JB; resources: SL and JB; data curation; PG and RM; writing—original draft preparation: PG and RM; writing—review and editing: PG, RM, SL, and JB; visualization: PG, RM, DN-B, SL, and JB; supervision: SL; project administration: SL; funding acquisition: SL and JB. All authors contributed to the article and approved the submitted version.

## Funding

This research was funded by NIH grants U01AI124260 and U01AI160421.

## Acknowledgments

We acknowledge Shabnam Jawahar and Natalia Kim, former research technicians at the Department of Molecular Parasitology in NYBC who helped in the maintenance of mouse BMDC cultures. We acknowledge Kathy Tang, Head of the LARS facility at NYBC for providing animal and veterinary care. The authors also thank Mihaela Barbu-Stevanovic, Head of the Flowcytometry Core facility at NYBC. The authors also acknowledge Maria Elena Bottazzi and Bin Zhan from Baylor College of Medicine, Texas Children’s Hospital Center for Vaccine Development, Houston, Texas for producing the recombinant rASP-1 proteins used in this study. We thank the members of the Genome Technology Core at JAX-GM for performing the RNAseq sequencing.

## Conflict of interest

The authors declare that the research was conducted in the absence of any commercial or financial relationships that could be construed as a potential conflict of interest.

## Publisher’s note

All claims expressed in this article are solely those of the authors and do not necessarily represent those of their affiliated organizations, or those of the publisher, the editors and the reviewers. Any product that may be evaluated in this article, or claim that may be made by its manufacturer, is not guaranteed or endorsed by the publisher.

## Supplementary material

Supplementary Figure 1 PAM3CSK4-activated murine CD11c^+^ BMDCs secrete significantly increased levels of IL-12p40 and TNF-α in a MyD88-dependent manner. Murine CD11c^+^ BMDCs were stimulated for 24 hr with Pam3CSK4 (1 µg/ml) in the presence or absence of MyD88X (50 µM). Unstimulated condition (media) served as control. Culture supernatants were analyzed for **(A)** IL-12p40 and **(B)** TNF-α. Data represents mean + SEM of 2-4 individual experiments. Each individual experiment had at least two technical replicates. MyD88X: MyD88 inhibitor. Statistical analysis was performed using Mann Whitney U test for unpaired ‘t test’ between and Wilcoxon-signed rank test for paired t test between presence and absence of inhibitors. ** P <0.01, **** P <0.0001.

Supplementary Figure 2 Representative FACS plots of differentiation of naïve CD4^+^ T cells into Th1, Tfh-like, Tfh1 and Th17 cells in the presence of activated BMDCs. Activated BMDCs were co-cultured with naïve CD4^+^ T cells for 4 days in 1:5 ratio. αCD3+ αCD28 stimulated naïve CD4^+^ T cells alone served as the positive control. Representative FACS plots of CD4^+^ T cells expressing IFN-γ, IL-21 and IL-17A. Percentage of differentiated T cells that express cytokines associated with Th1 cells (IFNγ ^+^), Tfh-like cells (IL-21^+^), Tfh1 cells co-expressing IFN-γ and IL-21 (IFNγ^+^IL-21^+^), Th17 cells (IL-17A^+^), and Tfh17 cells co-expressing IL-17A and IL-21 (IL-17A^+^IL-21^+^) as determined by flow cytometry analysis. MyD88X: MyD88 inhibitor, BX795: TRIF inhibitor.

Supplementary Figure 3 PAM3CSK4-activated murine BMDCs differentiated naïve CD4^+^ T cells to Th1 and Tfh-like cells in MyD88-dependent manner. PAM3CSK4-activated BMDCs were co-cultured with naïve CD4^+^ T cells for 4 days in 1:5 ratio. Percentage of differentiated T cells that express cytokines associated with **(A)** Th1 cells (IFNγ ^+^), **(B)** Tfh-like cells (IL-21^+^), **(C)** Tfh1 cells co-expressing IFN-γ and IL-21 (IFNγ^+^IL-21^+^), **(D)** Th17 cells (CD4^+^IL-17A^+^), and **(E)** Tfh17 cells co-expressing IL-17A and IL-21 (IL-17A^+^IL-21^+^) as determined by flow cytometry analysis. Data represents mean + SEM of two individual experiments. Each individual experiment had three technical replicates. MyD88X: MyD88 inhibitor. Statistical analysis was performed using Mann Whitney U test for unpaired ‘t test’ between and Wilcoxon-signed rank test for paired t test between presence and absence of inhibitors. * P <0.05, ** P <0.01.

Supplementary Figure 4 Representative FACS plots of differentiation of naïve CD4^+^ T cells into Th1, Tfh-like, Tfh1, and Th17 cells in the presence of rASP-1-activated BMDCs from WT and IFNAR^-/-^ mice. Activated BMDCs from **(A)** wild type (WT) mice and **(B)** type I IFN receptor deficient mice (IFNAR^-/-^) were co-cultured with naïve CD4^+^ T cells from WT mice for 4 days. Representative FACS plots of CD4^+^ T cells expressing IFN-γ and IL-21, IL-17A and IL-21 in WT and IFNAR^-/-^ mice was determined by flow cytometry analysis. WT: wild type C57BL/6J mice, IFNAR^-/-^: IFNAR-deficient mice of B6 background.

Supplementary Figure 5 Gating strategy to identify CD11c^+^ cDC cells in PBMCs.

Supplementary Figure 6 rASP-1-stimulated cDCs are distinct from the controls. Principle Component Analysis plots based on total genes (n=11,415; **A**), IFN genes (n=169; **B**) or Cytokine-associated genes (n=213; **C**). Gene lists used in **(B)** and **(C)** can be found in **Supplementary Table 1**.

Supplementary Figure 7 rASP-1 stimulation induced the up-regulation of genes associated with TLR signaling. Genes involved in TLR signaling **(A)**, or TLR4-TRAF signaling pathway **(B)** are shown using IPA. Shades of red: upregulated genes, shades of blue: down regulated genes, red line: prediction relationships that lead to activation, blue line: prediction relationships that lead to inhibition, yellow line: inconsistent prediction, grey line: effect not predicted.

Supplementary Figure 8 rASP-1 stimulation induced genes associated with DC maturation and their crosstalk with NK cells.

Supplementary Figure 9 rASP-1 stimulation induced genes associated with STAT signaling. Genes enriched in STAT3 **(A)**, IL-17 **(B)** and IL-21**(C)** signaling pathways are represented.

Supplementary Figure 10 Representative FACS plot of proliferation and differentiation of naïve human CD4^+^ T cells into Th- and Tfh-like cells in the presence of activated cDCs. Activated cDC-mediated proliferation and differentiation of naïve CD4^+^ T cells into **(A)** Th- (CD45RA^-^CXCR5^-^) and Tfh-like (CD45RA^-^CXCR5^+^) using three independent activated cDC samples were analyzed by flow cytometry. **(B)** Frequencies of Tfh-like cells, frequencies of IFN-γ^+^ (Th1), IL-17^+^ (Th17), IL-21^+^ (Tfh-like) and IL-13 (Th2) and cells in unstimulated (medium), rASP-1- or MPLA-stimulated co-culture assay.

Supplementary Figure 11 IgG and its subtypes IgG1 and IgG2c and not IgM antibody responses are critical in the rASP-1-adjuvanted IIV3 vaccine mediated protection of mice from H1N1 challenge. Mice were immunized i.m. with IIV3 or rASP-1-adjuvanted IIV3 vaccine (IIV3 + rASP-1) in WT, TLR4^-/-^, TRIF^-/-^, IFNAR^-/-^ and AID^-/-^ mice. Anti-IIV3 **(A)** IgG (end point titer) and its subtypes **(B)** IgG1 and **(C)** IgG2c antibody responses (endpoint titer) 2 weeks after primary immunization were detected by ELISA. **(D)** Anti-IIV3 IgM (O.D at 1:100 sera dilution) and **(E)** anti-IIV3 IgG (end point titer) antibody responses 2 weeks after primary immunization in WT versus AID^-/-^ mice. Titer of antibodies or O.D values are represented as mean ± SEM. Data in WT, TRIF^-/-^, TLR4^-/-^, IFNAR^-/-^ mice experiments represent 4-5 mice per immunization group from two individual experiments. While data in AID^-/-^ mice experiment represents 3-5 mice per group from one individual experiment. Significance was calculated using and unpaired, two-tailed Student’s t-test. **p <0.01 and ***p <0.001.

Supplementary Table 1 Differentially expressed genes (DEG) in rASP-1-activated human cDCs versus media alone.
