# Supplementary figures and images for: The Th1/Tfh-like biased responses elicited by the rASP-1 innate adjuvant are dependent on TRIF and Type I IFN receptor pathways
Source: Front Immunol. 2022 Sep 2;13:961094. doi: 10.3389/fimmu.2022.961094 (PMC9478378; doi:10.3389/fimmu.2022.961094)

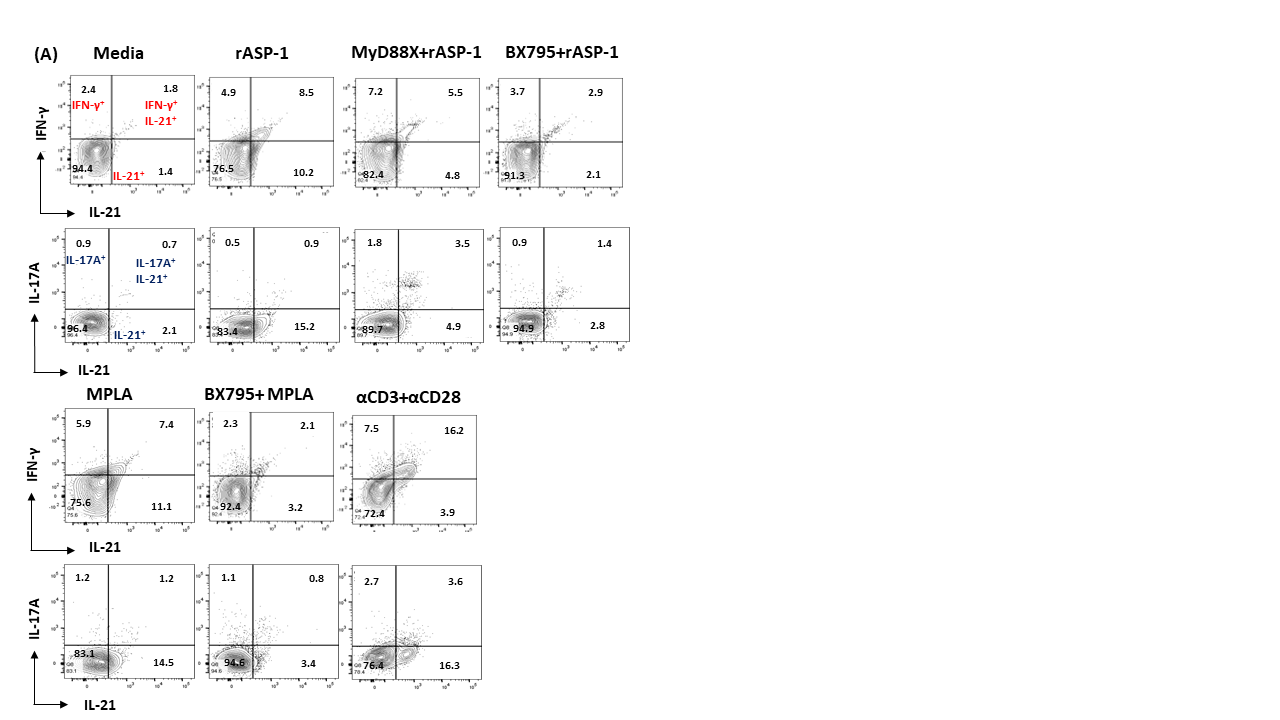

Supplement: Supplementary Figure 2 — Representative FACS plots of differentiation of naïve CD4+ T cells into Th1, Tfh-like, Tfh1 and Th17 cells in the presence of activated BMDCs. Activated BMDCs were co-cultured with naïve CD4+ T cells for 4 days in 1:5 ratio. αCD3+ αCD28 stimulated naïve CD4+ T cells alone served as the positive control. Representative FACS plots of CD4+ T cells expressing IFN-γ, IL-21 and IL-17A. Percentage of differentiated T cells that express cytokines associated with Th1 cells (IFNγ +), Tfh-like cells (IL-21+), Tfh1 cells co-expressing IFN-γ and IL-21 (IFNγ+IL-21+), Th17 cells (IL-17A+), and Tfh17 cells co-expressing IL-17A and IL-21 (IL-17A+IL-21+) as determined by flow cytometry analysis. MyD88X: MyD88 inhibitor, BX795: TRIF inhibitor. [file Image_2.tif]

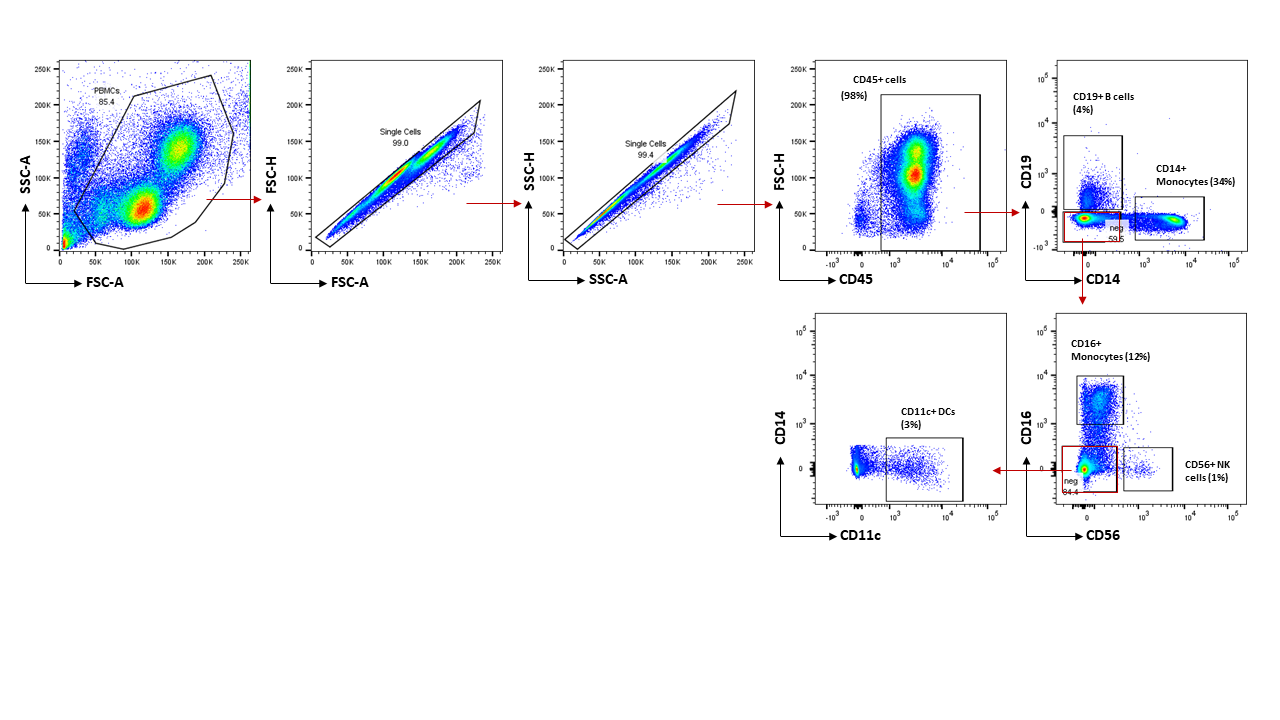

Supplement: Supplementary Figure 5 — Gating strategy to identify CD11c+ cDC cells in PBMCs. [file Image_5.tif]

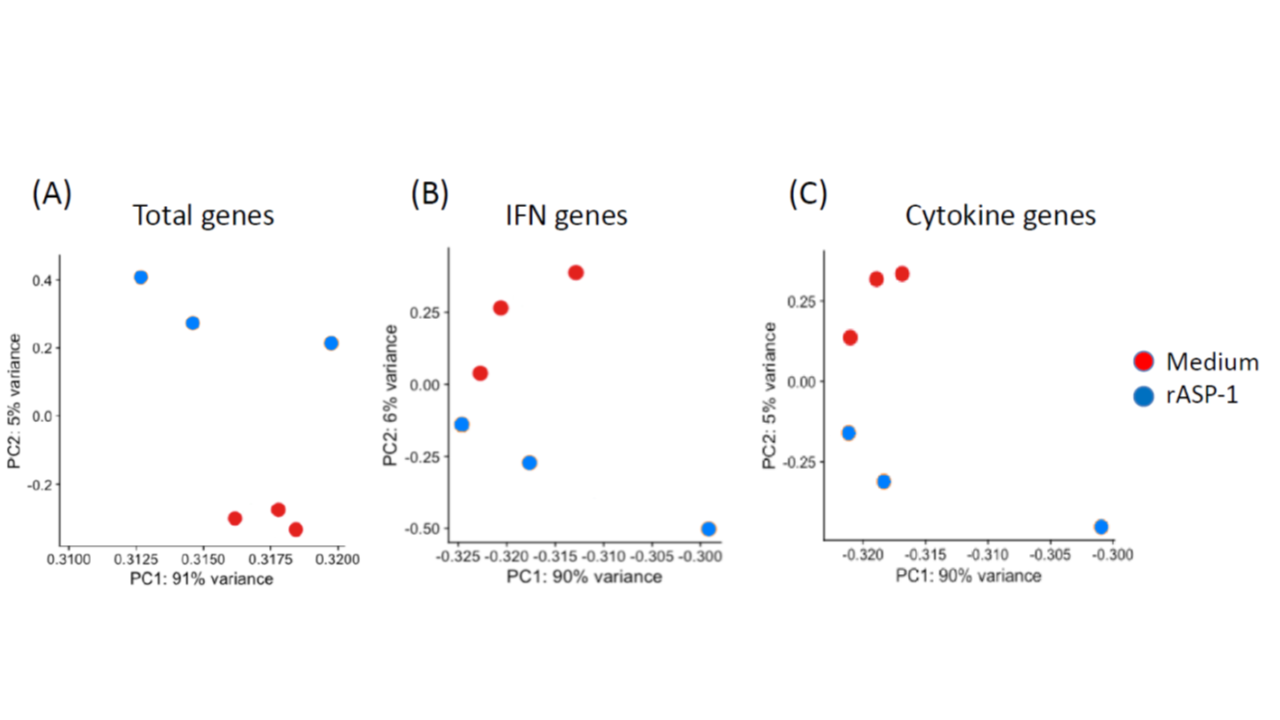

Supplement: Supplementary Figure 6 — rASP-1-stimulated cDCs are distinct from the controls. Principle Component Analysis plots based on total genes (n=11,415; A), IFN genes (n=169; B) or Cytokine-associated genes (n=213; C). Gene lists used in (B) and (C) can be found in Supplementary Table 1 . [file Image_6.tif]

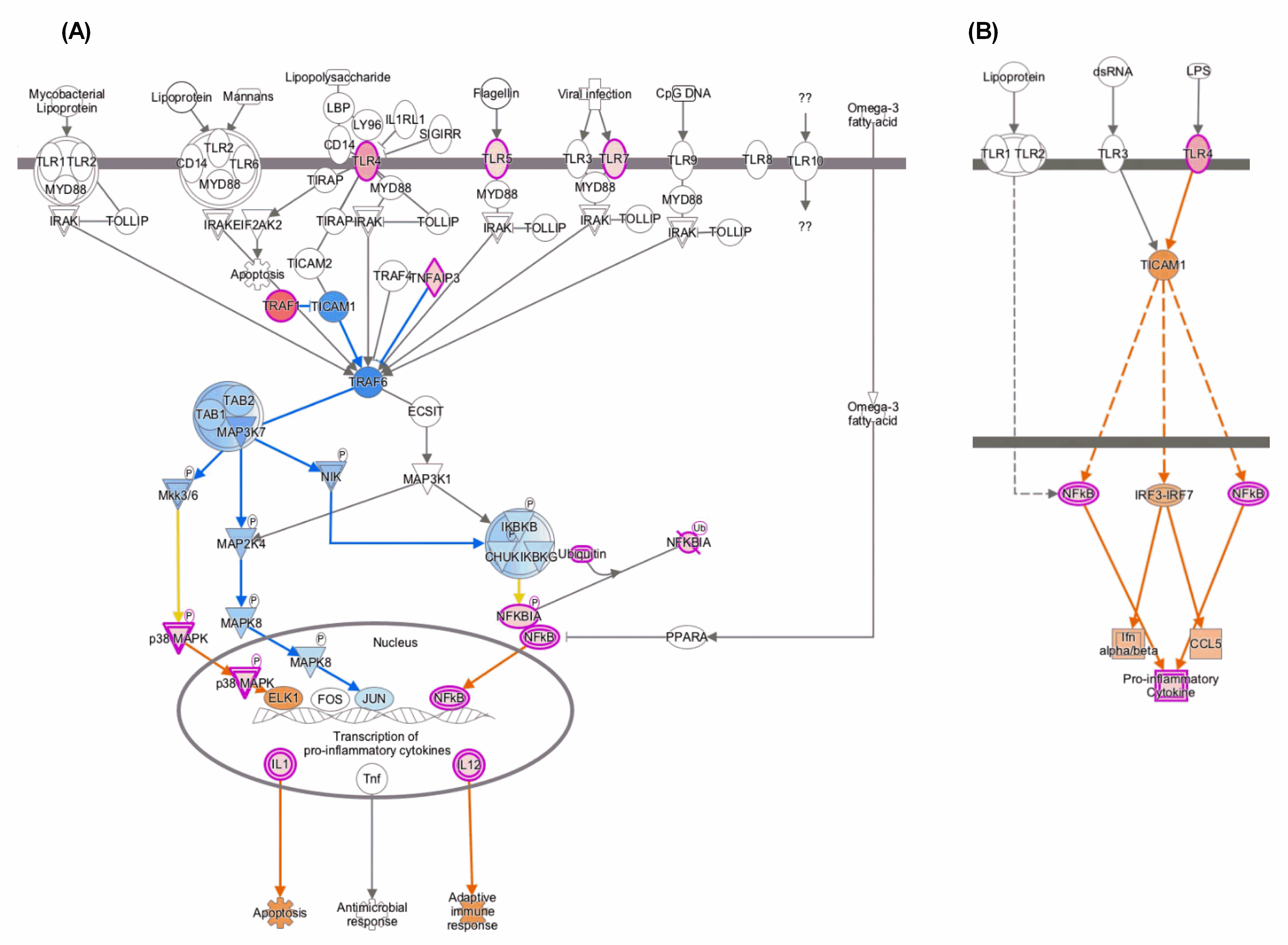

Supplement: Supplementary Figure 7 — rASP-1 stimulation induced the up-regulation of genes associated with TLR signaling. Genes involved in TLR signaling (A), or TLR4-TRAF signaling pathway (B) are shown using IPA. Shades of red: upregulated genes, shades of blue: down regulated genes, red line: prediction relationships that lead to activation, blue line: prediction relationships that lead to inhibition, yellow line: inconsistent prediction, grey line: effect not predicted. [file Image_7.tif]

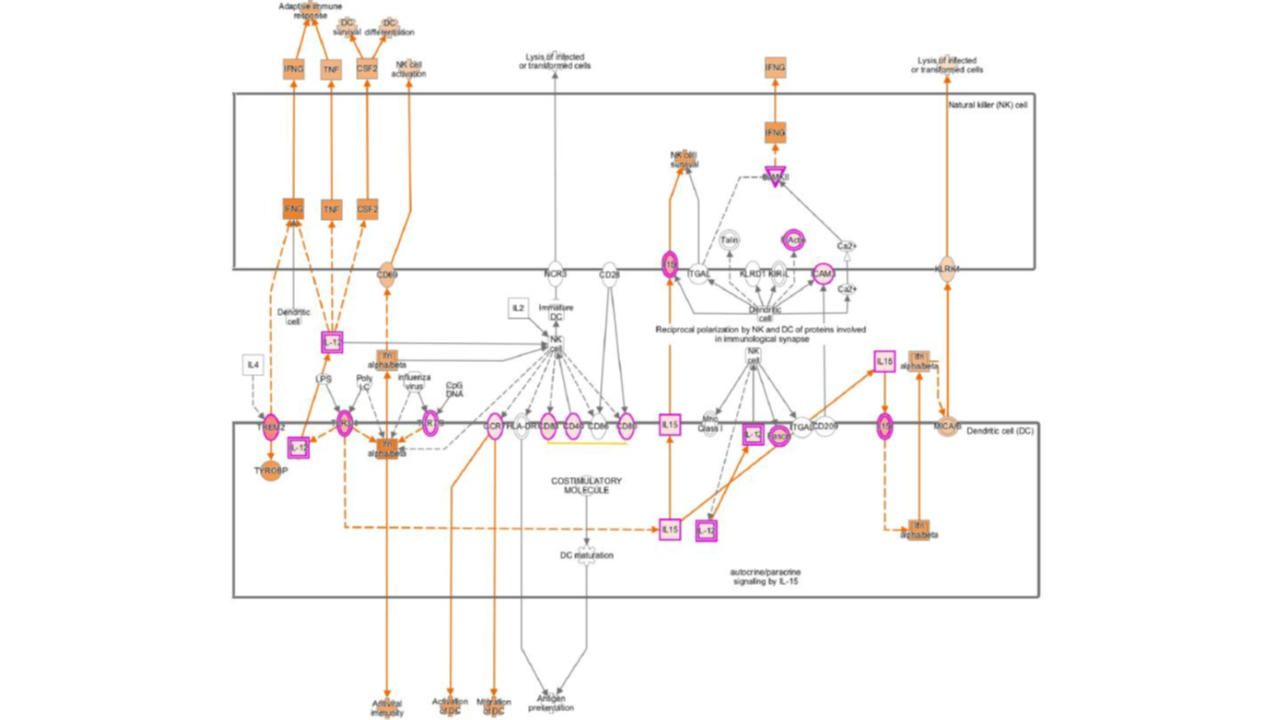

Supplement: Supplementary Figure 8 — rASP-1 stimulation induced genes associated with DC maturation and their crosstalk with NK cells. [file Image_8.tif]

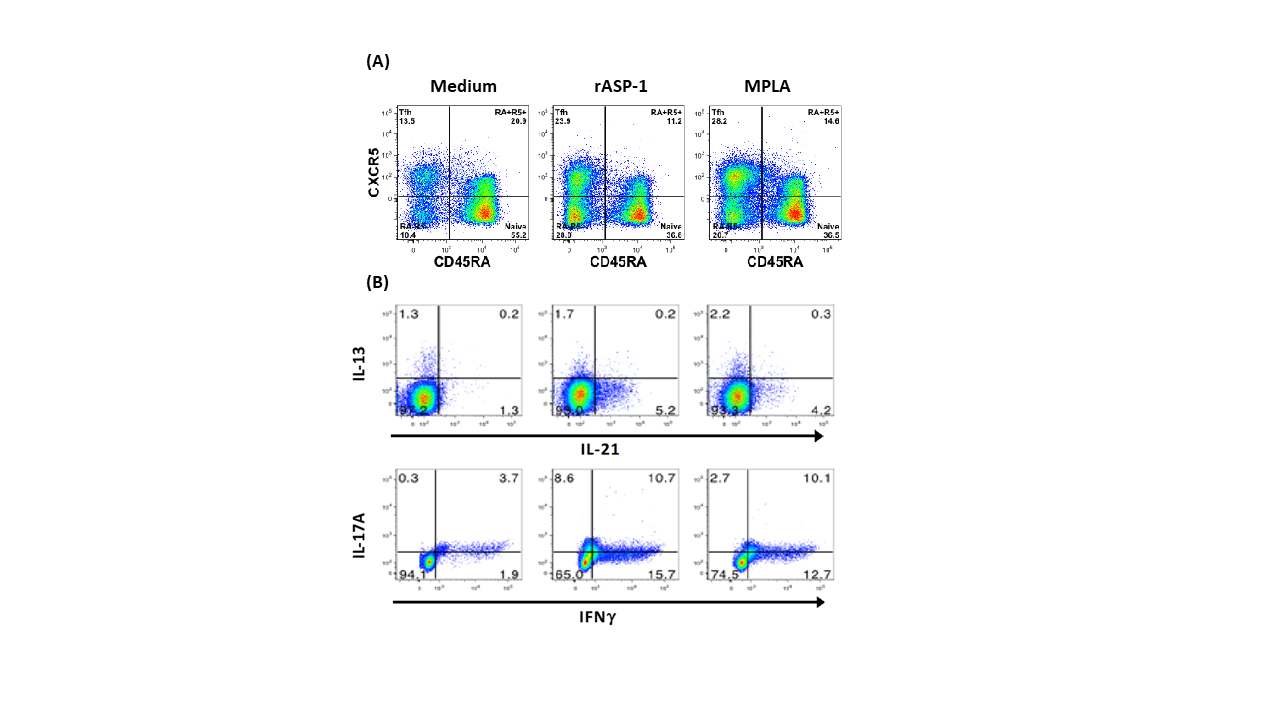

Supplement: Supplementary Figure 10 — Representative FACS plot of proliferation and differentiation of naïve human CD4+ T cells into Th- and Tfh-like cells in the presence of activated cDCs. Activated cDC-mediated proliferation and differentiation of naïve CD4+ T cells into (A) Th- (CD45RA-CXCR5-) and Tfh-like (CD45RA-CXCR5+) using three independent activated cDC samples were analyzed by flow cytometry. (B) Frequencies of Tfh-like cells, frequencies of IFN-γ+ (Th1), IL-17+ (Th17), IL-21+ (Tfh-like) and IL-13 (Th2) and cells in unstimulated (medium), rASP-1- or MPLA-stimulated co-culture assay. [file Image_10.tif]
